# Crystal structure of a cadmium sulfate coordination polymer based on the 3,6-bis­(pyrimidin-2-yl)-1,4-di­hydro-1,2,4,5-tetra­zine ligand

**DOI:** 10.1107/S2056989020006830

**Published:** 2020-05-29

**Authors:** Suwadee Jiajaroen, Kittipong Chainok

**Affiliations:** a Thammasat University Research Unit in Multifunctional Crystalline Materials and Applications (TU-MCMA), Faculty of Science and Technology,, Thammasat University, Khlong Luang, Pathum Thani, 12121, Thailand

**Keywords:** crystal structure, coordination polymers, cadmium(II), bis-bidentate coordination mode, tetra­zine

## Abstract

The polymeric title compound, poly[aqua­hemi[μ_2_-3,6-bis­(pyrimidin-2-yl)-1,4-di­hydro-1,2,4,5-tetra­zine](μ_3_-sulfato)­cadmium(II)], [Cd(SO_4_)(C_10_H_8_N_8_)_0.5_(H_2_O)]_*n*_, (**I**), represents an example of a three-dimensional coordination polymer resulting from the reaction of CdSO_4_·8/3H_2_O with 3,6-bis­(pyrimidin-2-yl)-1,4-di­hydro-1,2,4,5-tetra­zine (H_2_bmtz, C_10_H_8_N_8_) under hydro­thermal conditions. The Cd^II^ atom has a distorted octa­hedral coordination environment defined by two nitro­gen atoms from one H_2_bmtz ligand, three oxygen atoms from three different sulfate anions, and one oxygen atom from a coordinating water mol­ecule. The 1,4-di­hydro-1,2,4,5-tetra­zine ring of the H_2_bmtz ligand is located about an inversion center, with the NH group being equally disordered over two sites. The sulfate anion acts as a μ_3_-bridging ligand to connect three Cd^II^ atoms, resulting in the formation of [Cd(SO_4_)(H_2_O)] sheets propagating parallel to the *bc* plane. Adjacent sheets are inter­connected across the H_2_bmtz ligands, which coordinate the Cd^II^ atoms in a bis-bidentate coordination mode, to form a three-dimensional framework structure. The framework is further stabilized by classical O—H⋯O hydrogen bonds involving the coordinating water mol­ecules and the sulfate groups, and by N—H⋯O hydrogen bonds between the disordered tetra­zine NH groups and sulfate oxygen atom, along with C—H⋯π and π–π stacking [centroid-to-centroid separation = 3.5954 (15) Å] inter­actions between parallel pyrimidine rings of the H_2_bmtz ligand.

## Abstract 2

In the title compound, polymeric (100) sheets consisting of [Cd(SO_4_)(H_2_O)] units are linked by H_2_bmtz [H_2_bmtz is 3,6-bis­(pyrimidin-2-yl)-1,4-di­hydro-1,2,4,5-tetra­zine] into a three-dimensional structure.

## Chemical context   

Coordination polymers (CPs) are a class of organic–inorganic hybrid materials formed from metal ions or metal clusters and organic linkers through covalent bonds. The structural organization of CPs can result in chains, sheets or three-dimensional frameworks (Batten *et al.*, 2009 ▸). These hybrid materials have received extensive attention over the past three decades owing to their structural features and useful applications in the fields of gas storage and separation, catalysis, chemical sensing, magnetism or proton conduction (Furukawa *et al.*, 2010 ▸; Ye & Johnson, 2016 ▸; Espallargas & Coronado, 2018 ▸; Xu *et al.*, 2016 ▸; Zhang *et al.*, 2017 ▸). Nowadays, many multi-dimensional CPs with structural and topological diversity have been synthesized through the tremendous possibilities of choices for building blocks, and some of them seem promising as candidate materials, for instance, in gas purification (Duan *et al.*, 2015 ▸). In the context of the crystal engineering of CPs, the most feasible strategy for the construction of such infinite hybrid networks is by the careful selection of metal coordin­ation arrangements and suitable organic linkers. Among the most common ligands, the rigid organic carboxyl­ate- and pyridyl-based ligands have by far been the most widely used to control the structural motifs of these solids (Glöckle *et al.*, 2001 ▸).
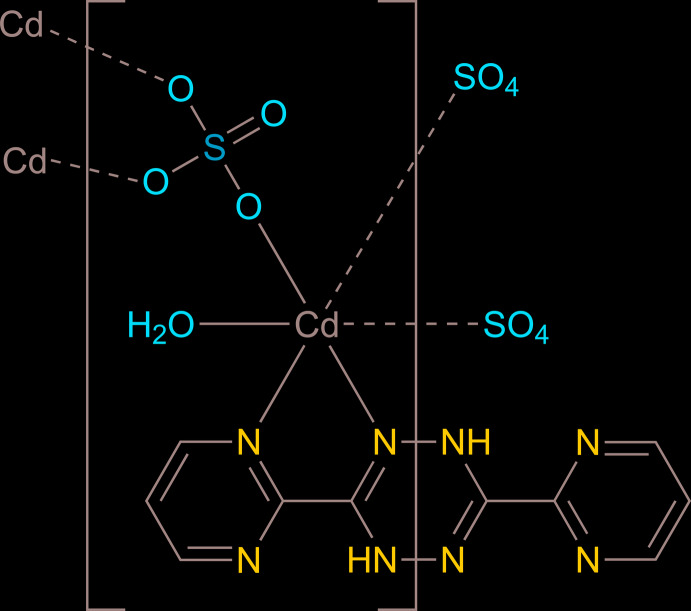


In this work, to explore the synthesis of novel CPs using 3,6-bis­(pyrimidin-2-yl)-1,4-di­hydro-1,2,4,5-tetra­zine, C_10_H_8_N_8_ or H_2_bmtz (Kaim & Fees, 1995 ▸; Chainok *et al.*, 2012 ▸) as a polydentate nitro­gen-donor ligand with cadmium(II) sulfate, a new CP [Cd(SO_4_)(H_2_bmtz)_0.5_(H_2_O)]_*n*_ (**I**) was isolated under hydro­thermal conditions. The crystal structure and supra­molecular inter­actions of (**I**) are reported herein.

## Structural commentary   

**Figure 1:**
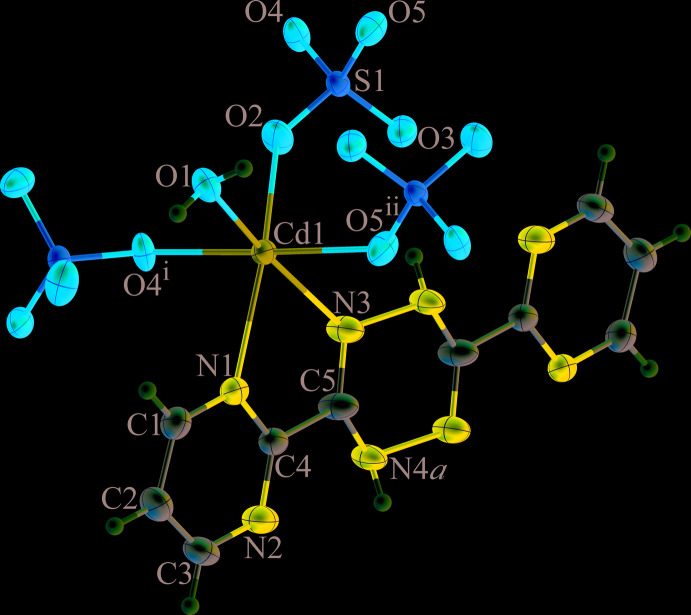
Mol­ecular structure of (**I**), showing the atom-labelling scheme. Only one orientation of the disordered N4—H group is shown. Displacement ellipsoids are drawn at the 50% probability level. [Symmetry codes: (i) 1 − *x*, −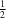 + *y*, 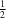 − *z*; (ii) 1 − *x*, 1 − *y*, 1 − *z*].

**Figure 2:**
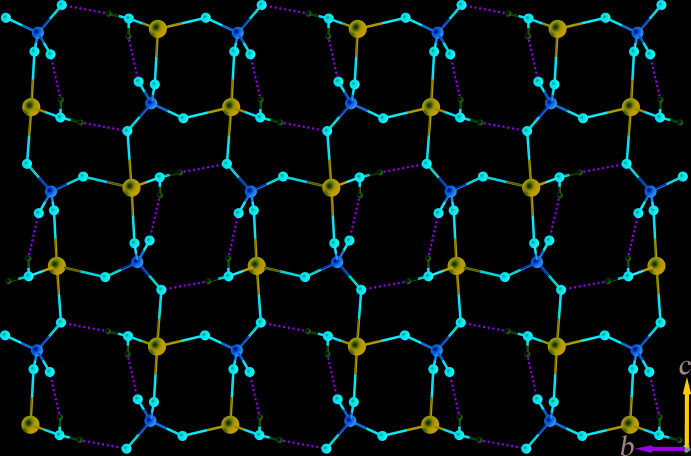
View of the [Cd(SO_4_)(H_2_O)] sheet in (**I**) propagating parallel to the *bc* plane. Classical O—H⋯O hydrogen-bonding inter­actions are shown as dashed lines.

**Figure 3:**
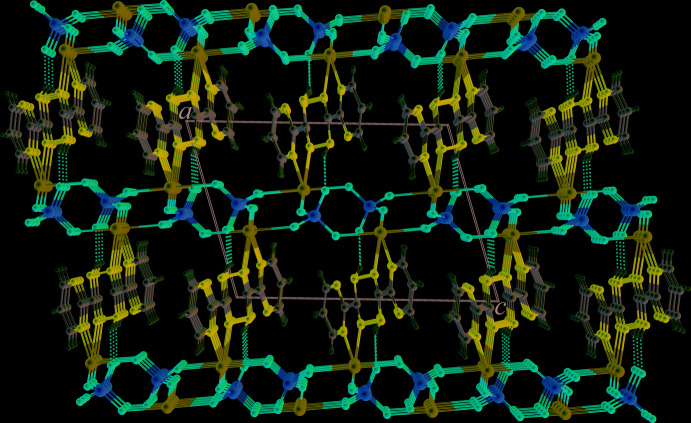
Packing diagram of (**I**), showing N—H⋯O hydrogen bonding as dashed lines.

The asymmetric unit of the title compound consists of one Cd^II^ cation, one-half of the H_2_bmtz ligand, one sulfate anion and one coordinating water mol­ecule. The 1,4-di­hydro-1,2,4,5-tetra­zine ring of the H_2_bmtz ligand is located about an inversion centre, with the NH group (N4) being equally disordered over two sites. As shown in Fig. 1 ▸, the Cd^II^ atom exhibits a distorted octa­hedral [CdN_2_O_4_] coordination environment with two nitro­gen atoms from the H_2_bmtz ligand, three oxygen atoms from three different sulfate anions and one oxygen atom from the coordinating water mol­ecule. The bond angles around the central Cd^II^ atom range from 69.69 (5) to 168.46 (5)°. The Cd—O and Cd—N bond lengths fall in the range of 2.2321 (12)–2.3790 (13) Å, which is comparable with those of reported cadmium(II) sulfate compounds containing additional nitro­gen donor ligands such as [Cd_2_(SO_4_)_2_(C_16_H_12_N_6_)_2_(H_2_O)_2_]·4H_2_O (GADLON; Harvey *et al.*, 2003 ▸), [Cd_2_(C_2_H_3_O_2_)_2_(S_2_O_8_)(C_15_H_11_N_2_)_2_(H_2_O)_2_]·7H_2_O (FOMBUF; Díaz de Vivar *et al.*, 2005 ▸) and [Cd_2_(C_15_H_9_N_9_)(H_2_O)_6_(SO_4_)_2_]·H_2_O (DIQCOX; Safin *et al.*, 2013 ▸). The complete H_2_bmtz mol­ecule is not planar (r.m.s. deviation = 0.111 Å) with the central six-membered ring of the 1,4-di­hydro-1,2,4,5-tetra­zine moiety in a twist-boat conformation; the C5—N3—N4*A* ^i^—C5^i^ torsion angle is 36.4 (4)° [symmetry code: (i) 2 − *x*, 1 − *y*, 1 − *z*]. The sulfate anion acts as a μ_3_-bridging ligand to connect three Cd^II^ atoms to form a sheet-like structure of [Cd(SO_4_)(H_2_O)] units, propagating parallel to the *bc* plane, Fig. 2 ▸. Adjacent sheets are inter­connected across the H_2_bmtz ligands, which exhibit a bis-bidentate coordination mode, giving rise to a three-dimensional framework structure, Fig. 3 ▸.

## Supra­molecular features   

**Figure 4:**
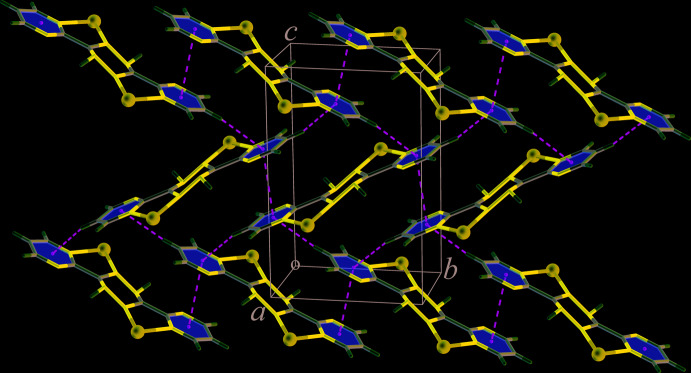
Partial packing diagram of (**I**), showing C—H⋯π and π–π stacking inter­actions (dashed lines) between the H_2_bmtz ligands.

In the crystal, classical O—H⋯O hydrogen bonds exist between the coordinating water mol­ecules and the sulfate groups, and N—H⋯O hydrogen bonds involving the disordered tetra­zine NH group and sulfate oxygen atoms. In this way, rings with 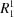
(8) and 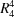
(16) graph-set motifs are formed, Table 1 ▸. Additionally, C—H⋯π [H⋯*Cg* = 3.34 (2) Å; *Cg* is the centroid of the pyrimidine ring] and π–π stacking [centroid-to-centroid separation = 3.5954 (15) Å, slippage between parallel pyrimidine rings = 1.131 Å] inter­actions between the pyrimidine rings of the H_2_bmtz ligand are also observed, Fig. 4 ▸.

## Database survey   

A search of the Cambridge Structural Database (CSD version 5.41, November 2019 update; Groom *et al.*, 2016 ▸) gave only two hits for H_2_bmtz complexes with transition metals ions, *viz*. with Cu^I^ (QORNAM; Glöckle *et al.*, 2001 ▸) and Ag^I^ (ZASTAQ; Chainok *et al.*, 2012 ▸). In these structures, the coordination mode of the H_2_bmtz ligands is bis-bidentate through nitro­gen atoms.

## Synthesis and crystallization   

All reagents were of analytical grade and were used as received without further purification. The ligand 3,6-bis­(pyrimidin-2-yl)-1,4-di­hydro-1,2,4,5-tetra­zine was synthesized according to a literature method (Kaim & Fees, 1995 ▸). A mixture solution of CdSO_4_·8/3H_2_O (41.7 mg, 0.2 mmol) and the H_2_bmtz ligand (36.7 mg, 0.1 mmol) in water (5 ml) was added into a 15 ml Teflon-lined reactor, stirred at room temperature for 10 min, sealed in a stainless steel autoclave and placed in an oven. The mixture was heated to 383 K under autogenous pressure for 48 h, and then cooled down to room temperature. After filtration, brown block-shaped crystals were obtained in 80% yield (33.4 mg) based on the cadmium(II) source.

## Refinement   

Crystal data, data collection and structure refinement details are summarized in Table 2 ▸. Nitro­gen atom N4 of the 1,4-di­hydro-1,2,4,5-tetra­zine ring was found to be disordered about an inversion centre; restraints (SADI and RIGU with esd 0.001 Å^2^) were used for its refinement. All hydrogen atoms were found in difference-Fourier maps. H atoms attached to C atoms were refined in the riding-model approximation with C—H = 0.93 Å and *U*_iso_(H) = 1.2*U*_eq_(C). The H atoms bound to O or N atoms were refined with distance restraints of O—H = 0.84 ± 0.01 Å and N—H = 0.86 ± 0.01 Å and with *U*_iso_(H) = 1.5*U*_eq_(O) and 1.2*U*_eq_(N), respectively.

## Supplementary Material

Crystal structure: contains datablock(s) I. DOI: 10.1107/S2056989020006830/wm5546sup1.cif

Structure factors: contains datablock(s) I. DOI: 10.1107/S2056989020006830/wm5546Isup2.hkl

Supporting information file. DOI: 10.1107/S2056989020006830/wm5546Isup3.cdx

CCDC reference: 2004950

Additional supporting information:  crystallographic information; 3D view; checkCIF report

## Figures and Tables

**Table 1:** Hydrogen-bond geometry (Å, °) *Cg*1 is the centroid of the N1/N2/C1–C4 ring.

*D*—H⋯*A*	*D*—H	H⋯*A*	*D*⋯*A*	*D*—H⋯*A*
O1—H1*A*⋯O3^i^	0.84 (2)	1.92 (2)	2.710 (2)	159 (2)
O1—H1*B*⋯O4^ii^	0.84 (2)	1.91 (2)	2.743 (2)	174 (3)
N4*A*—H4*A*⋯O3^iii^	0.87 (2)	2.09 (2)	2.889 (3)	153 (2)
N4*B*—H4*A*⋯O3^iii^	0.87 (2)	2.09 (2)	2.828 (3)	143 (2)
C2—H2⋯*Cg*1^iv^	0.93	3.34 (2)	4.091 (3)	140 (2)

Symmetry codes: (i) 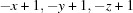; (ii) 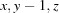; (iii) 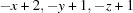; (iv) 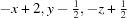.

**Table 2:** Experimental details

Crystal data	
Chemical formula	[Cd(SO_4_)(C_10_H_8_N_8_)_0.5_(H_2_O)]
*M*_r_	346.60
Crystal system, space group	Monoclinic, *P*2_1_/*c*
Temperature (K)	296
*a*, *b*, *c* (Å)	9.3000 (3), 7.9798 (2), 13.2586 (4)
β (°)	106.872 (1)
*V* (Å^3^)	941.60 (5)
*Z*	4
Radiation type	Mo *K*α
μ (mm^−1^)	2.56
Crystal size (mm)	0.28 × 0.24 × 0.18

Data collection	
Diffractometer	Bruker D8 QUEST CMOS
Absorption correction	Multi-scan (*SADABS*; Bruker, 2016 ▸)
*T*_min_, *T*_max_	0.660, 0.746
No. of measured, independent and observed [*I* > 2σ(*I*)] reflections	24280, 2357, 2338
*R*_int_	0.020
(sin θ/λ)_max_ (Å^−1^)	0.668

Refinement	
*R*[*F* ^2^ > 2σ(*F* ^2^)], *wR*(*F* ^2^), *S*	0.014, 0.037, 1.12
No. of reflections	2357
No. of parameters	167
No. of restraints	11
H-atom treatment	H atoms treated by a mixture of independent and constrained refinement
Δρ_max_, Δρ_min_ (e Å^−3^)	0.41, −0.37

Computer programs: *APEX3* and *SAINT* (Bruker, 2016 ▸), *SHELXT* (Sheldrick, 2015*a* ▸), *SHELXL* (Sheldrick, 2015*b* ▸) and *OLEX2* (Dolomanov *et al.*, 2009 ▸).

## supplementary crystallographic information

### Crystal data

**Table:**

[Cd(SO_4_)(C_10_H_8_N_8_)_0.5_(H_2_O)]	*F*(000) = 672
*M**_r_* = 346.60	*D*_x_ = 2.445 Mg m^−^^3^
Monoclinic, *P*2_1_/*c*	Mo *K*α radiation, λ = 0.71073 Å
*a* = 9.3000 (3) Å	Cell parameters from 9940 reflections
*b* = 7.9798 (2) Å	θ = 3.4–28.4°
*c* = 13.2586 (4) Å	µ = 2.56 mm^−^^1^
β = 106.872 (1)°	*T* = 296 K
*V* = 941.60 (5) Å^3^	Block, brown
*Z* = 4	0.28 × 0.24 × 0.18 mm

### Data collection

**Table:**

Bruker D8 QUEST CMOS diffractometer	2357 independent reflections
Radiation source: sealed x-ray tube	2338 reflections with *I* > 2σ(*I*)
Graphite monochromator	*R*_int_ = 0.020
Detector resolution: 7.39 pixels mm^-1^	θ_max_ = 28.4°, θ_min_ = 3.2°
φ and ω scans	*h* = −12→12
Absorption correction: multi-scan (*SADABS*; Bruker, 2016)	*k* = −10→10
*T*_min_ = 0.660, *T*_max_ = 0.746	*l* = −17→17
24280 measured reflections	

### Refinement

**Table:**

Refinement on *F*^2^	Hydrogen site location: mixed
Least-squares matrix: full	H atoms treated by a mixture of independent and constrained refinement
*R*[*F*^2^ > 2σ(*F*^2^)] = 0.014	*w* = 1/[σ^2^(*F*_o_^2^) + (0.0176*P*)^2^ + 0.6173*P*] where *P* = (*F*_o_^2^ + 2*F*_c_^2^)/3
*wR*(*F*^2^) = 0.037	(Δ/σ)_max_ = 0.002
*S* = 1.12	Δρ_max_ = 0.41 e Å^−^^3^
2357 reflections	Δρ_min_ = −0.37 e Å^−^^3^
167 parameters	Extinction correction: SHELXL-2018/3 (Sheldrick 2015b), Fc^*^=kFc[1+0.001xFc^2^λ^3^/sin(2θ)]^-1/4^
11 restraints	Extinction coefficient: 0.0013 (3)
Primary atom site location: dual	

### Special details

**Table:**

Geometry. All esds (except the esd in the dihedral angle between two l.s. planes) are estimated using the full covariance matrix. The cell esds are taken into account individually in the estimation of esds in distances, angles and torsion angles; correlations between esds in cell parameters are only used when they are defined by crystal symmetry. An approximate (isotropic) treatment of cell esds is used for estimating esds involving l.s. planes.

### Fractional atomic coordinates and isotropic or equivalent isotropic displacement parameters (Å^2^)

**Table:**

	*x*	*y*	*z*	*U*_iso_*/*U*_eq_	Occ. (<1)
Cd1	0.61850 (2)	0.31497 (2)	0.37692 (2)	0.01977 (5)	
S1	0.46994 (4)	0.71617 (4)	0.38900 (3)	0.01921 (8)	
O1	0.40176 (15)	0.17176 (15)	0.34420 (10)	0.0290 (2)	
H1A	0.378 (3)	0.170 (3)	0.4004 (13)	0.046 (7)*	
H1B	0.408 (3)	0.0706 (15)	0.329 (2)	0.049 (7)*	
O2	0.49134 (16)	0.55557 (15)	0.34172 (10)	0.0389 (3)	
O3	0.61350 (13)	0.77844 (18)	0.45697 (10)	0.0327 (3)	
O4	0.40858 (15)	0.83595 (14)	0.30158 (9)	0.0276 (2)	
O5	0.35993 (14)	0.70011 (18)	0.44827 (9)	0.0338 (3)	
N1	0.81264 (15)	0.12667 (17)	0.37110 (10)	0.0254 (3)	
N2	1.07599 (16)	0.1093 (2)	0.39678 (13)	0.0342 (3)	
N3	0.85731 (15)	0.44436 (17)	0.44639 (13)	0.0321 (3)	
N4A	1.1157 (3)	0.4194 (4)	0.4718 (3)	0.0326 (6)	0.5
H4A	1.180 (2)	0.345 (3)	0.5064 (15)	0.070 (9)*	
N4B	1.1132 (3)	0.3849 (4)	0.5340 (3)	0.0320 (6)	0.5
C1	0.7954 (2)	−0.0244 (2)	0.32666 (14)	0.0337 (4)	
H1	0.699820	−0.071019	0.303463	0.040*	
C2	0.9168 (2)	−0.1135 (3)	0.31425 (17)	0.0427 (4)	
H2	0.904140	−0.217907	0.281657	0.051*	
C3	1.0573 (2)	−0.0420 (3)	0.35194 (17)	0.0410 (4)	
H3	1.140880	−0.100588	0.345972	0.049*	
C4	0.95337 (18)	0.18608 (18)	0.40350 (13)	0.0243 (3)	
C5	0.97378 (19)	0.3543 (2)	0.45278 (17)	0.0354 (4)	

### Atomic displacement parameters (Å^2^)

**Table:**

	*U*^11^	*U*^22^	*U*^33^	*U*^12^	*U*^13^	*U*^23^
Cd1	0.01945 (7)	0.02157 (7)	0.01864 (7)	0.00090 (3)	0.00607 (4)	0.00089 (3)
S1	0.02314 (16)	0.01938 (15)	0.01601 (15)	0.00267 (12)	0.00708 (12)	0.00085 (12)
O1	0.0335 (6)	0.0267 (6)	0.0300 (6)	−0.0058 (4)	0.0145 (5)	−0.0068 (4)
O2	0.0544 (8)	0.0226 (6)	0.0341 (6)	0.0142 (5)	0.0042 (6)	−0.0037 (5)
O3	0.0235 (5)	0.0464 (7)	0.0285 (6)	−0.0025 (5)	0.0081 (5)	−0.0066 (5)
O4	0.0458 (7)	0.0195 (5)	0.0182 (5)	0.0052 (5)	0.0102 (5)	0.0032 (4)
O5	0.0249 (6)	0.0580 (8)	0.0200 (5)	−0.0031 (5)	0.0090 (4)	0.0059 (5)
N1	0.0235 (6)	0.0253 (6)	0.0262 (6)	0.0015 (5)	0.0053 (5)	−0.0010 (5)
N2	0.0250 (6)	0.0319 (7)	0.0456 (8)	0.0029 (6)	0.0101 (6)	−0.0042 (6)
N3	0.0208 (6)	0.0213 (6)	0.0537 (9)	−0.0026 (5)	0.0101 (6)	−0.0076 (6)
N4A	0.0192 (11)	0.0255 (14)	0.0529 (19)	−0.0017 (10)	0.0102 (12)	−0.0135 (14)
N4B	0.0227 (12)	0.0210 (13)	0.0506 (18)	−0.0004 (10)	0.0079 (11)	−0.0084 (13)
C1	0.0292 (8)	0.0321 (8)	0.0356 (9)	−0.0019 (7)	0.0025 (7)	−0.0077 (7)
C2	0.0421 (10)	0.0341 (9)	0.0480 (11)	0.0047 (8)	0.0067 (8)	−0.0178 (8)
C3	0.0331 (9)	0.0400 (10)	0.0498 (11)	0.0091 (8)	0.0119 (8)	−0.0117 (8)
C4	0.0235 (7)	0.0233 (7)	0.0262 (7)	0.0011 (5)	0.0072 (6)	0.0009 (5)
C5	0.0203 (7)	0.0228 (7)	0.0613 (11)	−0.0022 (6)	0.0087 (7)	−0.0069 (7)

### Geometric parameters (Å, º)

**Table:**

Cd1—O1	2.2472 (12)	N2—C4	1.320 (2)
Cd1—O2	2.2321 (12)	N3—N4A^iii^	1.505 (3)
Cd1—O4^i^	2.3122 (11)	N3—N4B^iii^	1.399 (3)
Cd1—O5^ii^	2.2708 (12)	N3—C5	1.282 (2)
Cd1—N1	2.3674 (13)	N4A—H4A	0.871 (10)
Cd1—N3	2.3790 (13)	N4A—C5	1.372 (3)
S1—O2	1.4653 (12)	N4B—H4A	0.866 (10)
S1—O3	1.4640 (12)	N4B—C5	1.445 (3)
S1—O4	1.4828 (11)	C1—H1	0.9300
S1—O5	1.4654 (12)	C1—C2	1.384 (3)
O1—H1A	0.835 (10)	C2—H2	0.9300
O1—H1B	0.839 (10)	C2—C3	1.379 (3)
N1—C1	1.331 (2)	C3—H3	0.9300
N1—C4	1.340 (2)	C4—C5	1.481 (2)
N2—C3	1.335 (2)		
			
O1—Cd1—O4^i^	90.81 (5)	C4—N2—C3	116.51 (15)
O1—Cd1—O5^ii^	88.77 (5)	N4A^iii^—N3—Cd1	122.32 (13)
O1—Cd1—N1	108.71 (5)	N4B^iii^—N3—Cd1	127.25 (15)
O1—Cd1—N3	168.46 (5)	C5—N3—Cd1	117.26 (11)
O2—Cd1—O1	90.34 (5)	C5—N3—N4A^iii^	113.35 (17)
O2—Cd1—O4^i^	80.21 (5)	C5—N3—N4B^iii^	114.67 (17)
O2—Cd1—O5^ii^	98.25 (5)	N3^iii^—N4A—H4A	99.5 (18)
O2—Cd1—N1	154.41 (5)	C5—N4A—N3^iii^	110.9 (2)
O2—Cd1—N3	94.94 (5)	C5—N4A—H4A	108.3 (19)
O4^i^—Cd1—N1	82.54 (5)	N3^iii^—N4B—H4A	108.1 (19)
O4^i^—Cd1—N3	100.19 (5)	N3^iii^—N4B—C5	112.9 (2)
O5^ii^—Cd1—O4^i^	178.41 (4)	C5—N4B—H4A	102.7 (18)
O5^ii^—Cd1—N1	99.05 (5)	N1—C1—H1	119.3
O5^ii^—Cd1—N3	80.32 (5)	N1—C1—C2	121.38 (16)
N1—Cd1—N3	69.69 (5)	C2—C1—H1	119.3
O2—S1—O4	107.38 (7)	C1—C2—H2	121.2
O2—S1—O5	110.83 (9)	C3—C2—C1	117.63 (17)
O3—S1—O2	110.33 (8)	C3—C2—H2	121.2
O3—S1—O4	109.74 (8)	N2—C3—C2	121.52 (17)
O3—S1—O5	110.69 (7)	N2—C3—H3	119.2
O5—S1—O4	107.78 (7)	C2—C3—H3	119.2
Cd1—O1—H1A	106.8 (18)	N1—C4—C5	116.76 (14)
Cd1—O1—H1B	114.6 (17)	N2—C4—N1	126.64 (15)
H1A—O1—H1B	105 (2)	N2—C4—C5	116.60 (15)
S1—O2—Cd1	142.41 (8)	N3—C5—N4A	123.30 (19)
S1—O4—Cd1^iv^	131.02 (7)	N3—C5—N4B	120.8 (2)
S1—O5—Cd1^ii^	133.11 (8)	N3—C5—C4	118.78 (15)
C1—N1—Cd1	126.47 (11)	N4A—C5—C4	114.68 (18)
C1—N1—C4	116.31 (14)	N4B—C5—C4	117.02 (18)
C4—N1—Cd1	116.63 (10)		
			
Cd1—N1—C1—C2	170.31 (15)	N2—C4—C5—N3	169.24 (18)
Cd1—N1—C4—N2	−172.52 (14)	N2—C4—C5—N4A	8.9 (3)
Cd1—N1—C4—C5	8.10 (19)	N2—C4—C5—N4B	−31.3 (3)
Cd1—N3—C5—N4A	167.1 (2)	N3^iii^—N4A—C5—N3	40.7 (4)
Cd1—N3—C5—N4B	−150.1 (2)	N3^iii^—N4A—C5—C4	−160.0 (2)
Cd1—N3—C5—C4	8.5 (2)	N3^iii^—N4B—C5—N3	−38.9 (4)
O2—S1—O4—Cd1^iv^	−10.01 (13)	N3^iii^—N4B—C5—C4	162.2 (2)
O2—S1—O5—Cd1^ii^	103.95 (11)	N4A^iii^—N3—C5—N4A	−41.6 (4)
O3—S1—O2—Cd1	38.91 (17)	N4A^iii^—N3—C5—C4	159.9 (2)
O3—S1—O4—Cd1^iv^	109.93 (10)	N4B^iii^—N3—C5—N4B	39.5 (4)
O3—S1—O5—Cd1^ii^	−18.81 (14)	N4B^iii^—N3—C5—C4	−161.9 (2)
O4—S1—O2—Cd1	158.47 (14)	C1—N1—C4—N2	−0.8 (3)
O4—S1—O5—Cd1^ii^	−138.82 (10)	C1—N1—C4—C5	179.83 (16)
O5—S1—O2—Cd1	−84.06 (16)	C1—C2—C3—N2	−1.5 (3)
O5—S1—O4—Cd1^iv^	−129.45 (10)	C3—N2—C4—N1	0.9 (3)
N1—C1—C2—C3	1.5 (3)	C3—N2—C4—C5	−179.74 (18)
N1—C4—C5—N3	−11.3 (3)	C4—N1—C1—C2	−0.5 (3)
N1—C4—C5—N4A	−171.7 (2)	C4—N2—C3—C2	0.3 (3)
N1—C4—C5—N4B	148.1 (2)		

Symmetry codes: (i) −*x*+1, *y*−1/2, −*z*+1/2; (ii) −*x*+1, −*y*+1, −*z*+1; (iii) −*x*+2, −*y*+1, −*z*+1; (iv) −*x*+1, *y*+1/2, −*z*+1/2.

### Hydrogen-bond geometry (Å, º)

*Cg*1 is the centroid of the N1/N2/C1–C4 ring.

**Table:**

*D*—H···*A*	*D*—H	H···*A*	*D*···*A*	*D*—H···*A*
O1—H1*A*···O3^ii^	0.84 (2)	1.92 (2)	2.710 (2)	159 (2)
O1—H1*B*···O4^v^	0.84 (2)	1.91 (2)	2.743 (2)	174 (3)
N4*A*—H4*A*···O3^iii^	0.87 (2)	2.09 (2)	2.889 (3)	153 (2)
N4*B*—H4*A*···O3^iii^	0.87 (2)	2.09 (2)	2.828 (3)	143 (2)
C2—H2···*Cg*1^vi^	0.93	3.34 (2)	4.091 (3)	140 (2)

Symmetry codes: (ii) −*x*+1, −*y*+1, −*z*+1; (iii) −*x*+2, −*y*+1, −*z*+1; (v) *x*, *y*−1, *z*; (vi) −*x*+2, *y*−1/2, −*z*+1/2.
